# A Comparison of Severe Maternal Morbidity After Pre- and Periviable Premature Prelabor Rupture of Membranes in Multiple Gestations: Expectant Management versus Pregnancy Termination

**DOI:** 10.1055/a-2644-0279

**Published:** 2025-07-09

**Authors:** Courtney T. Connolly, Siwei Xie, Ethan Gough, Marika A. Toscano

**Affiliations:** 1 Department of Gynecology and Obstetrics, Johns Hopkins University School of Medicine, Baltimore, Maryland; 2 Johns Hopkins Bloomberg School of Public Health, Baltimore, Maryland; 3 Division of Maternal Fetal-Medicine, Department of Gynecology and Obstetrics, Johns Hopkins University School of Medicine, Baltimore, Maryland

**Keywords:** maternal morbidity, maternal complications, adverse outcomes, preterm prelabor rupture of membranes, multiple gestation

## Abstract

**Objective:**

To compare severe maternal morbidity (SMM) and mortality after pre- or periviable prelabor rupture of membranes (pPPROM) in multiple gestation pregnancies among individuals choosing expectant management (EM) or termination of pregnancy (TOP).

**Study Design:**

A retrospective cohort study of multiple gestations with pPPROM between 14 ^0/7^ and 23 ^6/7^ at five hospitals within a large health system from 2011 to 2024. The primary outcome was SMM by the Centers for Disease Control (CDCs), 21 indicators compared between the two cohorts. Continuous outcomes were compared with Mann–Whitney U tests. Chi-square/Fisher's exact tests were used for categorical outcomes. Data was analyzed using R.

**Results:**

Forty-five twin and 1 triplet gestations were included ( *n* = 37 EM, *n* = 9 TOP). There were no differences in gestational age at PPROM, age, race, and history of PPROM. There were no maternal deaths and no differences in chorioamnionitis, sepsis, ICU admission, blood loss, or hospital readmission. Seventy point three percent of patients undergoing EM experienced some form of maternal morbidity, and 27.0% experienced at least one CDC SMM indicator, but this was not different between groups.

**Conclusion:**

One in four individuals with multiple gestations undergoing EM of pPPROM experienced at least one adverse maternal outcome by CDC criteria. There were no significant differences identified between EM and TOP, likely due to the study's limited size.

**Key Points:**

- More than 70% of multiples with pPPROM electing EM experienced an adverse maternal outcome.
- There was no difference in maternal morbidity detected between EM and termination.
- One in four multiples with pPPROM electing EM had a severe adverse maternal outcome.
- Given disparities in access to termination, understanding maternal risks of pPPROM is critical.

Pre- and periviable prelabor preterm rupture of membranes (pPPROM) at less than 24 weeks of gestation complicates <1% of pregnancies. 1 2 Multiple gestation pregnancy is a risk factor for PPROM, and it is known that previable PPROM and preterm birth are more common in multiple gestations: 7 to 10% for twins versus 2 to 4% for singletons. 3

Management options for pPPROM include expectant management (EM) with the hope to continue the pregnancy to a viable gestational age prior to delivery or onset of pregnancy complications or termination of pregnancy (TOP) by immediate delivery via induction of labor or dilation and evacuation (D&E) procedure. Of patients that choose EM, it is thought that about 50% will deliver within 1 week, and approximately 70 to 80% may deliver within 2 to 5 weeks. 4 A unique subset of those who choose EM in multiple gestation pregnancies includes delayed interval deliveries.

Although data to inform discussions on neonatal outcomes after pPPROM with EM are increasingly available, there are few studies that examine maternal morbidity, and very limited data specific to multiple gestation pregnancies. Studies that have investigated pPPROM have either excluded multiple gestation pregnancies or been limited by very small sample sizes. 1 3 5 6 7 8 The existing studies have demonstrated that there is a high risk of maternal morbidity of EM compared to TOP, including risks of infection, sepsis, and even maternal death, with the composite maternal morbidity rate approaching 60%. 1 3 8 9 In one study that did include multiple gestations, twin gestations with pPPROM had a greater than five times higher odds of composite maternal morbidity, drawing attention to multiple gestations as potentially higher risk for these complications than singletons. 3

To our knowledge, no study has exclusively examined the maternal morbidity associated with EM of pPPROM in twin or higher-order multiple gestation pregnancies. Our study compares EM versus TOP in multiple gestation pregnancies to better inform risk counseling and maternal morbidity specific to multiple gestation pregnancies.

## Materials and Methods

This was a retrospective cohort study of pregnant individuals with a confirmed multiple gestation pregnancy aged 15 to 55 years old that experienced pPPROM in the absence of labor between 14 ^0/7^ and 23 ^6/7^ weeks. Subjects were ascertained from a population receiving prenatal care and delivering at one of five hospitals within a large health system in the mid-Atlantic between January 1, 2013, and November 16, 2024. All hospitals are university-affiliated with access to maternal–fetal medicine specialists. Initial management (EM or interruption of pregnancy) was determined by the patient's decision within 24 hours of diagnosis of membrane rupture. Ethical approval was obtained from IRB #00407497, and a waiver of written documentation of informed consent was provided.

Eligible subjects were identified by query of the health system's electronic medical record (EMR) system and International Classification of Diseases (ICD) Tenth revision codes (or their ICD-9 equivalents). Subjects with potential eligibility were identified using the ICD-10 code O42 for a diagnosis of Premature Rupture of Membranes. A review of EMR was then performed by a trained clinician to identify cases occurring at <24 weeks. The diagnosis of pPPROM was confirmed by membrane rupture defined by one or more of the following objective findings: (1) pooling of amniotic fluid in the posterior fornix, (2) nitrazine positive fluid, (3) ferning positive fluid, (4) oligohydramnios with an amniotic fluid index ≤5 cm or maximum vertical pocket ≤2 cm, or (5) positive intra-amniotic dye test. Multiple gestations with delayed interval deliveries were included.

Patients were excluded if they had a singleton pregnancy, missing delivery data, spontaneous delivery within 24 hours of pPPROM (ICD-10 O42.10), pregnancies complicated by major fetal urinary tract anomalies associated with anhydramnios such as renal agenesis or severe lower urinary tract obstruction (ICD-10 O35.E), placenta accreta spectrum (ICD-10 O43.1), or any contraindication to EM. Exclusion by ICD-10 codes was performed first, followed by exclusion for variables not easily represented by ICD-10 codes using manual chart review by a trained clinician.

Because patients that underwent procedural termination via D&E procedure did not consistently have the ICD10 code for PPROM attached to their charts, a separate EMR query was run to extract all patients that underwent this procedure using current procedural terminology code 59841 and an operating room case code unique to our hospital system, as well as filtered by location on Labor and Delivery units. Manual chart review was then performed to identify all D&E procedures conducted for the indication of pPPROM.

Manual chart review was also performed to collect baseline maternal demographic data, obstetric history, delivery and/or procedural details, and outcomes.

Clinical management of pPPROM was determined by the admitting obstetrician and/or maternal–fetal medicine consultant through shared decision-making with comprehensive options counseling corresponding to clinical presentation. State laws during the study period allowed for pregnancy interruption up to fetal viability and at any time during the pregnancy if necessary to protect the health or life of the pregnant person or in the case of severe fetal abnormality. Hospital policies for management of pPPROM in the study's health system are aligned with the recommendations from the current Society for Maternal–Fetal Medicine guidelines. 2

In general, patients were not required to be admitted during the previable period if they opted to undergo EM. If discharged, they were instructed to monitor for signs and symptoms of infection and continue outpatient follow-up with planned admission at viability for administration of antenatal corticosteroids and neonatal intensive care unit proximity. The decision of when to administer latency antibiotics was left up to the physician at the time of diagnosis. Patients who opted for TOP were given the option for induction of labor termination or D&E. Patients opting for D&E were transferred to two of the five hospitals that had clinicians trained in performing D&E procedures. In cases of delayed interval twin delivery, a high cord ligation of the umbilical cord of the delivered fetus was performed, and the patient remained admitted until delivery.

The primary outcome of the study was a composite measure of severe maternal morbidity (SMM) based on the Centers for Disease Control's (CDC) 21 indicators of maternal morbidity. 10 A secondary outcome included additional measures of SMM developed for this study based on current pPPROM literature. 1 3 5 11 This included chorioamnionitis (defined by (1) maternal fever (≥38.0°C) associated with one or more of the following: maternal leukocytosis, purulent cervical drainage, fetal tachycardia or (2) clinical suspicion in the absence of fever if other clinical signs were present), 12 13 endometritis (uterine fundal tenderness, fever ≥38.0°C with exclusion of other infection, and/or malodorous purulent lochia within 72 hours of delivery), sepsis (multiorgan dysfunction by a score ≥2 on the Sequential Organ Failure Assessment score), 14 and septic shock (sepsis criteria plus requiring vasopressors to maintain mean arterial pressure ≥65 with a lactic acid higher than 2 millimoles/L). 15

### Statistics

Descriptive statistics were generated to compare the baseline characteristics of the cohort, pPPROM outcomes, and maternal morbidity among patients with opted for EM versus TOP. The Wilcoxon rank-sum test was used to compare continuous variables. For categorical variables, Fisher's exact test was used. For all analyses, statistical significance was determined at α of 0.05. Data cleaning and all analyses were performed using R (version 4.4.2). This manuscript was prepared using the “strengthening the reporting of observational studies in epidemiology” (STROBE) criteria for observational studies. 16

## Results

**Fig. 1:**
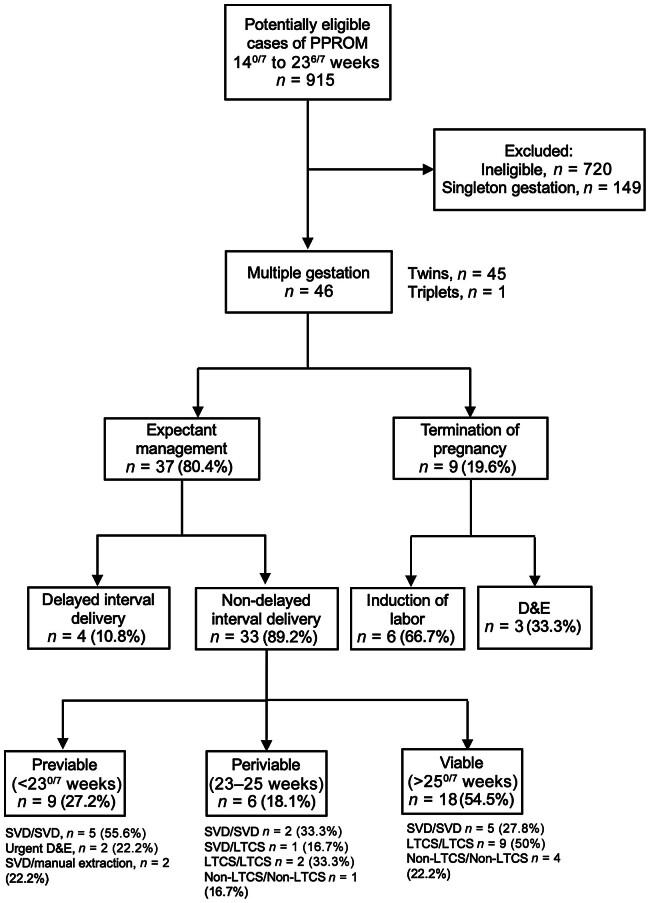
Study population flow diagram of patients with pre- and periviable prelabor preterm rupture of membranes with multiple gestation pregnancies between 14 ^0/7^ and 23 ^6/7^ . D&E, dilation and evacuation; LTCS, low-transverse cesarean section; non-LTCS, non-low transverse cesarean section; PPROM, previable preterm rupture of membranes; SVD, spontaneous vaginal delivery.

There were 915 cases identified as potential pPPROM less than 24 weeks of gestation based on the initial eligibility criteria. After applying inclusion and exclusion criteria and performing manual chart review, 46 multiple gestation pregnancies with pPPROM were included ( Fig. 1 ). Of these 46 patients, 37 opted for EM and 9 opted for interruption of pregnancy. There were 45 twin pregnancies (23 dichorionic–diamniotic, 20 monochorionic–diamniotic, and 2 monochorionic–monoamniotic) and 1 triplet pregnancy (with a monochorionic–diamniotic pair) in the cohort.

Table 1 presents the baseline characteristics of the study population. There were no significant differences in maternal age, Hispanic ethnicity, race, public insurance status, history of PPROM, history of uterine surgery, and history of preterm labor between the groups. There was a relatively high rate of iatrogenic pPPROM, 24.3% in the EM cohort and 44.4% in the TOP cohort, although not significantly different between the groups.

**Table 1:** Baseline characteristics of multiple gestations choosing expectant management and termination of pregnancy for pre- and periviable rupture of membranes

Outcome variable	Expectant management	Termination of pregnancy	*p* -Value
	*n* = 37	*n* = 9	
Maternal age (y)	32 (28–38)	31 (27–35)	0.8027
Hispanic	1 (2.8)	1 (11.1)	0.3636
Race			0.4368
Caucasian	18 (50.0)	7 (77.8)	
Black/African American	15 (41.7)	1 (11.1)	
Asian	1 (2.8)	0 (0.0)	
Native Hawaiian/Other Pacific Islander	0 (0.0)	0 (0.0)	
Other/multiracial	2 (5.6)	1 (11.1)	
Public insurance	8 (22.2)	1 (12.5)	1.0000
History of PPROM	2 (8.7)	0 (0.0)	1.0000
History of uterine surgery	25 (69.4)	5 (55.6)	0.4539
History of preterm labor	5 (21.7)	1 (12.5)	1.0000
History of preterm birth	5 (20.8)	3 (37.5)	0.3785
Pregnancy is a result of assisted reproductive technology	11 (31.4)	2 (22.2)	0.7030
Cerclage is in place during the current pregnancy	4 (10.8)	0 (0.0)	0.5715
Type 2 diabetes mellitus	1 (2.8)	0 (0.0)	1.0000
Chronic hypertension	4 (11.1)	3 (33.3)	0.1307
Iatrogenic PPROM	9 (24.3)	4 (44.4)	0.2462
Gestational age at PPROM diagnosis	21.3 (18.6–23.0)	19.4 (16.0–21.4)	0.1050

Note: All data presented as *n* (%) or median (IQR).

Table 2 presents the delivery outcomes for the EM cohort of fetuses 1 and 2. There were four cases of delayed interval delivery, all in dichorionic–diamniotic pregnancies, which account for the minor differences between fetuses 1 and 2. The median pregnancy latency period from pPPROM to delivery was 13 (IQR: 3–63) days for fetus 1 and 17 (3–67) days for fetus 2. The median gestational age at delivery was 24.7 (21.6–28.6) weeks for fetus 1 and 25.1 (22.3–29.2) weeks for fetus 2. The majority of deliveries occurred in the setting of preterm labor, 75.7% for fetus 1 and 67.6% for fetus 2. There were some combined multimodal deliveries with higher incidence of vaginal deliveries for fetus 1 compared with fetus 2 (51.4% vs. 40.5%) and higher incidence of low transverse and non-low transverse Cesarean sections for fetus 2 compared with fetus 1 (32.4 and 16.2% vs. 29.7 and 13.5%, respectively).

**Table 2:** Delivery outcomes for the expectant management cohort

Outcome variable	Multiple fetus 1; *n* = 37	Multiple fetus 2; *n* = 37
Gestational age at PPROM diagnosis (wk)	21.3 (18.6–23.0)	21.3 (18.6–23.0)
Pregnancy latency period from PPROM to delivery (d)	13 (3–63)	17 (3–67)
Gestational age at delivery (wk)	24.7 (21.6–28.6)	25.1 (22.3–29.2)
Periviability period at delivery (wk)		
<23 ^0/7^	14 (37.8)	12 (32.4)
23 ^0/7^ –24 ^6/7^	5 (13.5)	6 (16.2)
≥25 ^0/7^	18 (48.7)	19 (51.4)
Mode of delivery		
Vaginal delivery	19 (51.4)	15 (40.5)
Low transverse cesarean section	11 (29.7)	12 (32.4)
Non-low transverse cesarean section	5 (13.5)	6 (16.2)
Dilation and evacuation	2 (5.4)	2 (5.4)
Manual extraction in OR	0 (0.0)	2 (5.4)
Indication for delivery		
Chorioamnionitis	7 (18.9)	9 (24.3)
Nonreassuring fetal heart tracing	3 (8.1)	3 (8.1)
Preterm labor	28 (75.7)	25 (67.6)
In utero fetal demise	1 (2.7)	1 (2.7)
Iatrogenic delivery for PPROM at 34 wk	1 (2.7)	1 (2.7)
Other maternal indications	2 (5.4)	2 (5.4)
Other, not specified	3 (8.1)	3 (8.1)

Note: All data presented as *n* (%) or median (IQR).

Rates of SMM were high in both cohorts, 70.3% for those elective EM and 55.6% for those electing TOP ( Table 3 ). The most common severe morbidity experienced by patients undergoing EM was hemorrhage (50.0%), followed by chorioamnionitis (29.7%), unplanned operative procedure after delivery (21.6%), and need for blood transfusion (18.9%). The most common morbidity in the TOP cohort was unplanned operative procedure after delivery (44.4%), followed by hemorrhage (12.5%) and need for blood transfusion (11.1%). There were no incidences of endometritis, sepsis, septic shock, unplanned hysterectomy, and ICU admission in the TOP cohort, but these severe morbidities were all identified in the EM cohort.

**Table 3:** Maternal morbidity outcomes of multiple gestations choosing expectant management and termination of pregnancy for pre- and periviable rupture of membranes

Outcome variable	Expectant management	Termination of pregnancy	*p* -Value
	*n* = 37	*n* = 9	
Chorioamnionitis	11 (29.7)	0 (0.0)	0.0890
Endometritis	2 (5.4)	0 (0.0)	1.0000
Sepsis	3 (8.1)	0 (0.0)	1.0000
Septic shock	2 (5.4)	0 (0.0)	1.0000
Unplanned hysterectomy	2 (5.4)	0 (0.0)	1.0000
Unplanned hysterotomy (excluding delivery by cesarean section)	1 (2.7)	0 (0.0)	1.0000
Injury requiring repair during delivery	2 (5.4)	0 (0.0)	1.0000
Unplanned operative procedure after delivery	8 (21.6)	4 (44.4)	0.2114
D and C	5 (13.5)	4 (44.4)	–
Laparoscopy	0 (0.0)	0 (0.0)	
Laparotomy	1 (2.7)	0 (0.0)	
Other	3 (8.1)	0 (0.0)	
Hemorrhage (≥1,000 mL)	18 (50.0)	1 (12.5)	0.1111
Blood loss at type of delivery (mL)	1,000 (425–1,400)	800 (350–851)	0.0706
Blood transfusion	7 (18.9)	1 (11.1)	1.0000
ICU admission	2 (5.4)	0 (0.0)	1.0000
Acute renal insufficiency	1 (2.7)	0 (0.0)	1.0000
Venous thromboembolism	1 (2.7)	0 (0.0)	1.0000
Readmission to hospital within 6 wk of discharge	1 (2.8)	0 (0.0)	1.0000
Death	0 (0.0)	0 (0.0)	1.0000
Composite maternal morbidity index a	26 (70.3)	5 (55.6)	0.4453

Note: All data presented as *n* (%) or median (IQR).

a One or more of the above categorical variables present, excluding the variable “blood loss at delivery.”

An analysis of SMM by the CDC's criteria demonstrated a higher incidence of experiencing at least one of the CDC indicators of SMM in the EM cohort (27.0% vs. 11.1%, *p* = 0.4212). However, this finding was not statistically significant and in both cohorts was largely driven by the need for blood transfusion ( Table 4 ). There were no maternal deaths in either group.

**Table 4:** CDC SMM outcomes of multiple gestations choosing expectant management and termination of pregnancy for pre- and periviable rupture of membranes

Outcome variable	Expectant management	Termination of pregnancy	*p* -Value
	*n* = 37	*n* = 9	
Acute myocardial infarction	1 (2.7)	0 (0.0)	1.0000
Aneurysm	0 (0.0)	0 (0.0)	1.0000
Acute renal failure	1 (2.7)	0 (0.0)	1.0000
Acute respiratory distress syndrome	1 (2.7)	0 (0.0)	1.0000
Amniotic fluid embolism	0 (0.0)	0 (0.0)	1.0000
Cardiac arrest/ventricular fibrillation	0 (0.0)	0 (0.0)	1.0000
Conversion of cardiac rhythm	0 (0.0)	0 (0.0)	1.0000
Disseminated intravascular coagulation	0 (0.0)	0 (0.0)	1.0000
Blood transfusion	7 (18.9)	1 (11.1)	1.0000
Eclampsia	0 (0.0)	0 (0.0)	1.0000
Heart failure/arrest during surgery or procedure	0 (0.0)	0 (0.0)	1.0000
Puerperal cerebrovascular disorder	0 (0.0)	0 (0.0)	1.0000
Pulmonary edema/acute heart failure	3 (8.11)	0 (0.00)	1.0000
Heart failure	2 (5.4)	0 (0.0)	1.0000
Severe anesthesia complications	0 (0.0)	0 (0.0)	1.0000
Sepsis	3 (8.1)	0 (0.0)	1.0000
Shock	2 (5.4)	0 (0.0)	1.0000
Sickle cell disease with crisis	0 (0.00)	NA	–
Air and thrombotic embolism	1 (2.7)	0 (0.0)	1.0000
Hysterectomy	2 (5.4)	0 (0.0)	1.0000
Temporary tracheostomy	0 (0.0)	0 (0.0)	1.0000
Ventilation	1 (2.7)	0 (0.0)	1.0000
Composite severe maternal morbidity CDC index (at least one above variables met)	10 (27.0)	1 (11.1)	0.4212

Note: All data presented as *n* (%).

## Discussion

The principal finding of this study is the very high rate of maternal morbidity in general among individuals with multiple gestations and pPPROM. Although not statistically significant (likely due to limited sample size), there was a trend toward higher SMM in patients electing EM versus TOP. Furthermore, the subtypes of SMM occurring in patients electing EM varied widely, and included sepsis, septic shock, unplanned hysterectomy, intensive care unit admission, renal failure, and others, while subtypes of SMM occurring in those electing TOP was solely related to blood transfusion or unplanned return to operating room with the majority for uterine aspiration.

That there is maternal morbidity inherent to carrying a multiple gestation pregnancy is a well-established fact, with demonstrated higher risk of prenatal, intrapartum, and postpartum complications. 17 18 Twin pregnancies, in general, are associated with a four-fold increased risk for severe maternal complications compared to singletons and higher maternal mortality. 19 20 As a baseline comparator, Binstock et al identified that 2.4% of twin deliveries overall met criteria for SMM by the CDC's indicators, mainly due to postpartum hemorrhage with transfusion. 17 Rates of SMM by CDC's indicators in the current study greatly exceed this baseline, at 27% in the EM and 11.1% in the TOP cohorts. This suggests pPPROM amplifies the overall risk of SMM in an already at-risk multiple pregnancy.

This study's findings are aligned with the results of existing literature on maternal morbidity following pPPROM. Among a combined group of singletons and multiple gestations, Sklar et al demonstrated that individuals opting for EM experienced a significantly increased risk of maternal morbidity when compared to TOP, with composite maternal morbidity rates of 60.2 and 33.0% for TOP and EM, respectively. 1 Using the same composite maternal morbidity index, we identified similar SMM rates of 70.3 and 55.6% for TOP and EM, respectively, though our findings did not reach statistical significance as our sample size was more limited due to inclusion of multiple gestations alone.

Due to the rarity of this condition and limited ability to ascertain it at a population level, most of the current literature on pPPROM is limited to EHR-based cohort studies with small sample sizes, even more so for samples with multiple gestations. In fact, many studies exclude multiple gestations, perhaps because of hypothesized pathophysiological differences that are thought to influence PPROM in multiples versus singletons. The results presented in this current study highlight that maternal morbidity data for multiples may not accurately be reflected by generalizing results of studies with populations comprised of a majority of singleton pregnancies. Data specific to multiples is critically important to aid in clinical decision making and counseling for this patient population.

It is also important to note our population's relatively high rate of iatrogenic pPPROM, ranging from 24 to 44%. There are existing studies that have examined iatrogenic pPPROM after fetal interventions; however, these have predominantly focused on fetal outcomes with a lack of maternal morbidity data. 21 22 Iatrogenic pPPROM is unique in its mechanism, via mechanical rupture rather than an inflammatory event, and is known to occur more frequently in multiple gestation pregnancies. 23 This study draws attention to the complexity of pPPROM in multiple gestations and invites further research on maternal morbidity for this subset of patients as well.

Finally, as our study emphasizes the risk of adverse maternal outcomes with EM of pPPROM in multiple gestations, it also underscores the critical need for access to comprehensive reproductive healthcare. This is becoming increasingly important as changing legislation restricts access to pregnancy termination and can limit options for patients with medically complex, high-risk pregnancies. It has been shown that patients who live in states with abortion restrictions face higher risks of maternal death and morbidity, amplified further by racial and socioeconomic disparities. 24 25 26

### Strengths and Limitations

Study strengths include that the patient population is drawn from a large and diverse population derived from five hospitals with maternal–fetal medicine and neonatology services. Considering the rarity of our outcome, this allowed the creation of the largest series to date of 46 multiple gestation pregnancies with pPPROM, all receiving counseling and management in a standardized fashion due to unified health system policies. To our knowledge, no study has explicitly compared EM to TOP in multiple gestation pregnancies alone, and this investigation has not been done across multiple hospitals. However, our results may not be generalizable outside our health system or to other states with varying abortion restrictions.

Our study is inherently limited by its retrospective nature and overall small sample size. Given the sample size, we were underpowered to detect differences in maternal morbidity and adverse outcomes. Furthermore, because we used ICD-10 coding in our ascertainment strategy, it is possible that the same patients were unintentionally excluded due to miscoding, though by using multiple EHR query strategies, we endeavored to capture as many cases as possible.

## Conclusion

In conclusion, the majority of patients with multiple gestation pregnancies that experience pPPROM will experience some form of maternal morbidity, with close to one in four individuals electing for EM experiencing at least one severe adverse maternal outcome by CDC criteria. We were underpowered to detect any significant differences in maternal morbidity in individuals electing for EM versus TOP, but were able to identify a trend toward higher rates of severe morbidity, with a wider range of morbidity types, in those electing EM. Further research is needed to examine maternal morbidity among multiple gestation pregnancies experiencing pPPROM, as the current evidence base is insufficient for adequate risk counseling.
